# Morphology and Light‐Dependent Spatial Distribution of Spin Defects in Carbon Nitride

**DOI:** 10.1002/anie.202210640

**Published:** 2022-09-27

**Authors:** Arianna Actis, Michele Melchionna, Giacomo Filippini, Paolo Fornasiero, Maurizio Prato, Enrico Salvadori, Mario Chiesa

**Affiliations:** ^1^ Department of Chemistry and NIS Centre University of Torino Via Pietro Giuria 7 10125 Torino Italy; ^2^ Department of Chemical and Pharmaceutical, INSTM UdR University of Trieste Via Licio Giorgieri 1 34127 Trieste Italy; ^3^ ICCOM-CNR URT Trieste Italy; ^4^ Center for Cooperative Research in Biomaterials (CIC biomaGUNE) Basque Research and Technology Alliance (BRTA) Paseo Miramon 194 20014 Donostia San Sebastián Spain; ^5^ Basque Fdn Sci Ikerbasque 48013 Bilbao Spain

**Keywords:** Carbon Nitride, Dipolar Spectroscopy, EPR Spectroscopy, Photocatalysis, Pulsed EPR, Radicals

## Abstract

Carbon nitride (CN) is a heterogeneous photocatalyst that combines good structural properties and a broad scope. The photocatalytic efficiency of CN is associated with the presence of defective and radical species. An accurate description of defective states—both at a local and extended level—is key to develop a thorough mechanistic understanding of the photophysics of CN. In turn, this will maximise the generation and usage of photogenerated charge carriers and minimise wasteful charge recombination. Here the influence of morphology and light‐excitation on the number and chemical nature of radical defects is assessed. By exploiting the magnetic dipole‐dipole coupling, the spatial distribution of native radicals in CN is derived with high precision. From the analysis an average distance in the range 1.99–2.34 nm is determined, which corresponds to pairs of radicals located approximately four tri‐s‐triazine units apart.

## Graphical abstract

Radicals in carbon nitride are invoked as photocatalytically competent species. However, their structure and localisation is ill‐defined. Here we provide a quantitative assessment of speciation, electronic structure, long‐range distributions and photo‐response of such radicals, as a function of carbon nitride morphology. This work paves the way to the engineering of the defective structure of carbon nitride and related 2D materials.

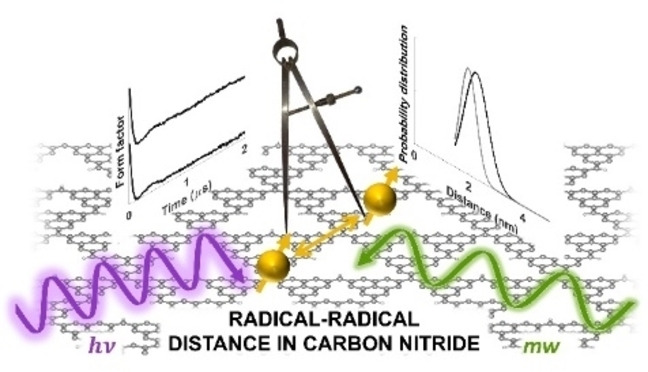

## Introduction

**Figure 1:**
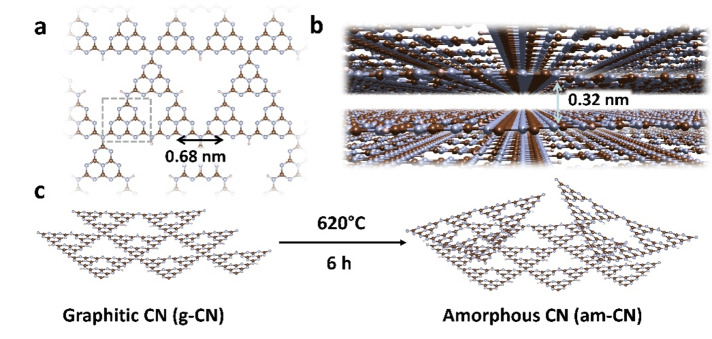
a) Top and b) side view of the 2D layered structure of carbon nitride (entry code: ICSD‐194746). The cell parameters are indicated by double‐headed arrows. In panel (a) the basic tri‐*s*‐triazine unit motif is highlighted by dashed lines. c) Schematic of the synthetic procedure used to generate amorphous CN from the graphitic form. Colour code: carbon (brown), nitrogen (blue), hydrogen (white).

Amongst the most promising photocatalysts, carbon nitride (CN) occupies a central place since it is a simple and robust semiconductor material with attractive features such as a metal‐free chemical composition, strong light absorption, tunable band edges, scalability and recyclability.[^1^, ^2^, ^3^] CN has been shown to be an active photocatalyst in H_2_ production,[^4^, ^5^] oxygen reduction,^[6]^ water oxidation and CO_2_ reduction[^7^, ^8^] as well as functionalization of arenes and heteroarenes with fluoroalkylated groups^[9]^ and C−C and C‐heteroatom coupling.^[10]^ In the most general description, CN has a graphite‐like structure that is held together by different types of bonds: strong, covalent bonds between the atoms of the polymeric triazine units, a series of hydrogen bonds that keep together the polymeric strands to form a 2D layer, and weaker van der Waals forces between adjacent layers.^[11]^ The basic motif constituting CN is the tri‐*s*‐triazine unit that corresponds to the ideal stoichiometry C_3_N_4_, although other elements such as H and O are typically present. X‐ray diffraction studies reveal that the in plane cell parameter is ≈0.68 nm while the distance between two adjacent layers is ≈0.32 nm, see Figure 1a, b. Owing to the extended π‐system, CN has a strong absorption in the near UV (≈380 nm), which tails off in the visible imparting a pale yellow colour to the material. In light of photocatalytic applications, strategies have been developed to extend and enhance the absorption properties of CN towards the visible. These range from red‐ox chemical treatments,^[12]^ to chemical doping with elements such as oxygen and sulphur[^13^, ^14^] or by decoration and functionalisation of the scaffold with side groups.^[15]^ However, all these modified forms of CN display an optical band gap spanning the region 2.50–2.75 eV and a similar C/N stoichiometric ratio.^[12]^ An interesting case, which is also a notable exception, is represented by thermally treated CN in Ar atmosphere. The treatment causes some degree of amorphization (Figure 1c), a higher C/N stoichiometric ratio and the appearance of an absorption band at ≈480 nm.[^12^, ^16^] Interestingly, transient absorption spectroscopy has identified lower‐energy trap states as responsible for the photoreactivity of CN.^[17]^ The involvement of trap states in redox process makes CN a flexible catalyst able to decouple oxidation and reduction events in the presence of reagents. However, the efficiency of light‐induced processes in CN is low and greatly limited by prompt fluorescence and electron/hole pair recombination.^[18]^ Such deactivation mechanisms are affected by the material morphology in a dual fashion. On one hand less crystalline materials often display a higher reactivity proportional to the number of surface states and defects, on the other hand the same surface states and defects promote charge recombination and decrease the global efficiency of the process.^[19]^ A comprehensive understanding of the mechanisms governing both the photophysics and the photochemistry of the materials thus entails a description of the defective states in terms of their concentration, local structure and spatial distribution, since all of these affect the response under light excitation and the chemistry that the material is able to drive. Characterisation techniques based on x‐ray absorption spectroscopy or nuclear magnetic resonance are extremely powerful in defining the structural and spatial aspects of compounds and materials, but lack the same capability for defective states.

This is because defects often occur in (very) low concentration and do not form ordered structures. Since numerous and relevant defects bear unpaired electrons, the latter offers a unique handle to define their local structure and pinpoint their relative location in space. To this respect, electron paramagnetic resonance (EPR) spectroscopy is unique as it allows the definition of the local structure of paramagnetic (radical) defects as well as their long‐range spatial distribution. The local structure can be derived by measuring hyperfine couplings, which quantify the interaction between the electron spin and the surrounding nuclear spins, whereas a convenient way to extract nanometre‐scale distances, specific to centres bearing unpaired electrons, is constituted by pulsed dipolar spectroscopy (PDS) which measures the magnetic dipole–dipole interaction between pairs of electron spins.[^20^, ^21^, ^22^] In this contribution, we assess the distribution of paramagnetic defective species in CN with sub‐nanometer precision and follow their evolution under light irradiation. To evaluate the effect of morphology, we consider CN prepared through two different methods that have been demonstrated to alter structural motives and affect the catalytic performances of the material. Previous studies on the perfluoroalkylation of electron‐rich organic substrates have suggested a correlation between number of paramagnetic defects and catalytic activity with a catalytic yield of <5% for the most crystalline CN as opposed to >99 % for the least crystalline. The reaction is thought to proceed through a radical mechanism initiated by photo‐induced charge separation within the semiconductor and charge transfer to the substrate adsorbed on the CN surface.^[12]^ Our analysis demonstrates that CN materials with different morphology stabilise distinct types of defects and that to higher crystallinity correspond a larger variety of paramagnetic defects. Furthermore, we determine an average distance between neighbouring paramagnetic defect sites spanning the range 1.99–2.34 nm. This value defines the spatial density of paramagnetic sites, a crucial factor in determining the turnover rates of chemical reactions promoted by paramagnetic centers.

## Results and Discussion

**Figure 2:**
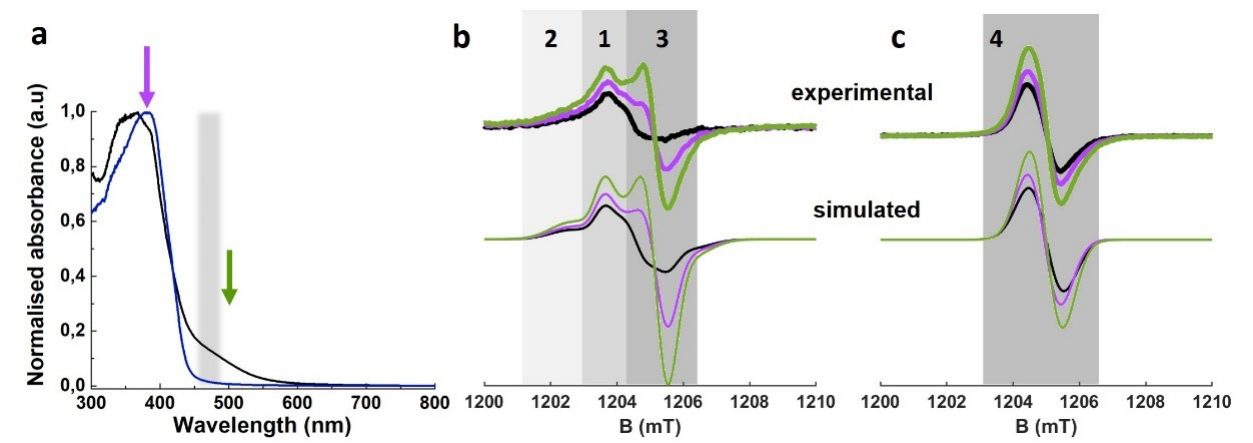
a) Normalised diffuse reflectance spectra for g‐CN (blue) and am‐CN (black) samples^[1]^ recorded at room temperature. The grey shaded area highlights the position of the band gap, whereas the arrows indicate the wavelength used to excite the sample either above (355 nm) or below (500 nm) the band gap. b,c) 33.8 GHz (Q‐band) CW EPR spectra recorded on g‐CN (b) and am‐CN (c) in dark (black lines) and after illumination at 355 nm (purple lines) and 500 nm (green lines). The corresponding simulations are reported underneath. The shaded areas highlight the spectral regions where each species dominates. All EPR spectra were recorded at 50 K.

In order to assess the photophysics of CN and how it is affected by morphological modification, two complementary materials were considered. g‐CN represents the pristine material with smooth sheet‐like geometry and an average crystallite size of 6.5±0.5 nm, whereas the thermally treated am‐CN represent a partially amorphous structure with a less regular geometry due to the disruption of the NH⋅⋅⋅N hydrogen bonds between polymeric strands induced by thermal treatment. In turn this reflects in a lower crystallite size of 5.5±0.5 nm, see Supplementary Figure S1. Figure 2a reports the UV/Vis spectra for g‐CN and am‐CN obtained through diffuse reflectance spectroscopy. The spectra of g‐CN and am‐CN differ in that the latter is characterised by an absorption maximum in the visible with a maximum at ≈500 nm and a long and weak absorption tail that extends beyond 700 nm. However, the band gap value estimated from the data (Tauc plot analysis) is only slightly reduced by the chemical treatment (2.71 and 2.52 eV for g‐CN and am‐CN, respectively), Figure 2a reports the average position of the spread of the band gap as a grey shaded area. Structural and optical data are in agreement with previous reports.^[12]^

We have previously reported a comparison of the photocatalytic activity of CN morphologies, including g‐CN and am‐CN, for the C−C coupling reaction between perfluoroalkyl compounds and aryl derivatives. The study showed the instrumental role of spin defects in am‐CN towards the considerably higher catalytic activity.^[12]^ Recent DFT calculations showed how defects, associated with intragap states, behave as photoactive sites that transfer electrons to the adsorbed perfluoroalkyl substrate upon visible light irradiation.^[23]^ EPR spectroscopy was employed to provide an insight into the origin of the altered absorption pattern of am‐CN and how structural morphology affect the fate of charge carriers after light excitation (trapped holes and electrons). This is of high significance for understanding rates of product formation during organo‐photocatalysis promoted by CN, also in recently developed dual‐catalytic systems.^[24]^ The paramagnetic (radical) species present in the material were investigated at low temperature in the dark and after irradiation below (500 nm and corresponding to the absorption maximum of am‐CN) or above (355 nm) the band gap. Figures 2b, c report the EPR spectra recorded at 33.8 GHz (Q‐band). The presence of radical species in carbon nitride is well‐known and has been extensively documented.[^25^, ^26^, ^27^, ^28^, ^29^] However, most EPR experiments have been conducted at 9.5 GHz (X‐band frequency) and lack sufficient spectral resolution. The higher microwave frequency used to record the spectra reported in Figure 2b, c allows for a higher resolution and demonstrates that presence of multiple species and an intricate response under photoexcitation. The EPR spectrum of g‐CN is composite and reveals the presence of at least three distinct species (the individual components are reported in the Supplementary Figure S2). Two of such species (**1**: *g*_iso_=2.0049, 1204.5 mT and **2**: *g*_iso_=2.0051, 1204.4 mT) are present in the dark and increase in intensity upon irradiation both at 355 and 500 nm, while the third (**3**: *g*_iso_=2.0037, 1205.2 mT) is only present after illumination, is more sensitive to excitation at 500 nm, has a tendency to recombine even at 50 K and promptly disappears after thermal annealing (for room temperature spectra see Supplementary Figure S3). On the other hand, the EPR spectrum of am‐CN (Figure 2c) can be accounted for by a single species (**4**: *g*_iso_=2.0042, 1204.9 mT), which increases in intensity after illumination regardless of the wavelength used. g‐CN and am‐CN display characteristic response to light irradiation. In particular, species **3** is only observed after illumination suggesting that in dark it corresponds to a diamagnetic state, either empty or doubly occupied. On the other hand, species **1**, **2** and **4** detected even in the dark exist as empty, singly and doubly occupied states. In fact, an increase in signal intensity after irradiation above the band gap (355 nm) stems from electron being promoted from the valence to the conduction band and eventually trapped in empty defective sites within the band gap. On the other hand, an increase in signal intensity after irradiation below the band gap (500 nm) is indicative of doubly occupied defects ionised to the conduction band and ultimately trapped in alike empty defects. The CW‐EPR data are supported also by pulsed experiments (see Figure 4), which confirm the same number of species and their behaviour after photoirradiation. Moreover, as previously reported by some of us,^[30]^ the EPR spectra in Figure 2 confirm that the absolute number of radicals present in am‐CN is larger than g‐CN, as shown by the different signal‐to‐noise ratio. To help identify each component and to increase the robustness of the assignment, both CW and pulsed spectra for each sample were simulated at once considering the same set of parameters, which are collected in Table 1, whereas the corresponding simulations are reported in Figure 2b, c. It is to note that pairs of radicals that are very close together may form EPR silent (antiferromagnetically coupled) species, that do not contribute to the observed signal.

**Table 1:** *g*‐values extracted from the simulations of Q‐band EPR spectra.

Material	Species	*g*_1_	*g*_2_	*g*_3_	*g*_iso_
g‐CN	1	2.0063±0.0002	2.0049±0.0003	2.0036±0.0004	2.0049
g‐CN	2	2.0080±0.0002	2.0050±0.0002	2.0023±0.0004	2.0051
g‐CN	3	–	–	–	2.0037±0.0003
am‐CN	4	–	–	–	2.0042±0.0003

**Figure 3:**
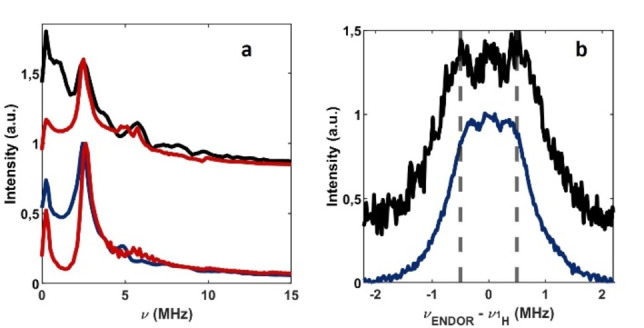
a) Experimental frequency domain two‐pulse ESEEM time traces recorded at 50 K for species **1** in g‐CN (black line) and species **4** in am‐CN (blue line). The corresponding simulations, based on the parameters reported in the main text, are displayed in red. b) ^1^H Mims ENDOR spectra for species **1** in g‐CN (black line) and species **4** in am‐CN (blue line). The vertical dashed lines are guides to the eye to highlight the different hyperfine couplings. The frequency scale on the *x*‐axis reports the deviation of the resonance lines from the Larmor frequency, *ν*_H_. Two‐pulse ESEEM and ^1^H ENDOR spectra were recorded at 1204.40 mT and 1204.75 mT for g‐CN and am‐CN, respectively.

An insight into the local electronic and geometric structure of the paramagnetic centers detected in CN is provided by two‐pulse Electron Spin Echo Envelope Modulation (ESEEM) and Electron Nuclear Double Resonance (ENDOR) spectroscopies which detect the interaction between the electron spin and the surrounding nuclear spins of ^14^N (*I*=1) and ^1^H (*I*=1/2), respectively. Figure 3 reports the ESEEM and ENDOR spectra recorded for species **1** and **4** at 50 K. From simulation of the ESEEM spectra (Figure 3a) a hyperfine coupling of *a*_iso_^N^=0.9±0.2 MHz, a dipolar component *T* ^N^=0.8±0.2 MHz and a nuclear quadrupole coupling *e* ^2^ *qQ*/*h*=3.0±0.2 MHz can be estimated. The same values are estimated for species **2** and **3**, see Supplementary Figures S4 and S5. The small measured ^14^N hyperfine coupling suggests that the electron spin is strongly delocalised over the tri‐*s*‐triazine structural motif of CN and not localised, for instance, on the edge of the layer. As a point of reference, the ^14^N hyperfine coupling measured in this study can be compared to the value for molecular radicals such as CN⋅^[31]^ and H_2_CN⋅^[32]^ for which an *a*_iso_^N^ of 12 MHz and 25 MHz have been reported. Such values are at least an order of magnitude larger than the largest hyperfine coupling measured in this work, indicating a strong delocalisation of the unpaired electron wavefunction in CN materials. This is further confirmed by ^1^H ENDOR spectra that report a maximum hyperfine coupling of 1.0 and 0.7 MHz for **1** and **4**, respectively (see Figure 3b). Interestingly, also radical **3** that appears only after irradiation displays similar hyperfine values indicating a similar degree of delocalisation. In the point‐dipole approximation this establishes a lower limit for the electron spin—H nuclear spin distance of 4.3 Å (see Supplementary Figure S6). Despite the limitation of the point‐dipole approximation for such a delocalised system, the value compares favourably with the distance of ≈3.8–4.5 Å between the center of the tri‐*s*‐triazine unit and the closest ^1^H derived from the crystal structure. Further support to the localisation of the radical on a single tri‐*s*‐triazine unit is provided by a DFT study on plausible radical defects in CN.^[23]^ This can be understood considering that adjacent tri‐*s*‐triazine units are linked by sp^3^ hybrid Ns, which serve as isolating linkers.

**Figure 4:**
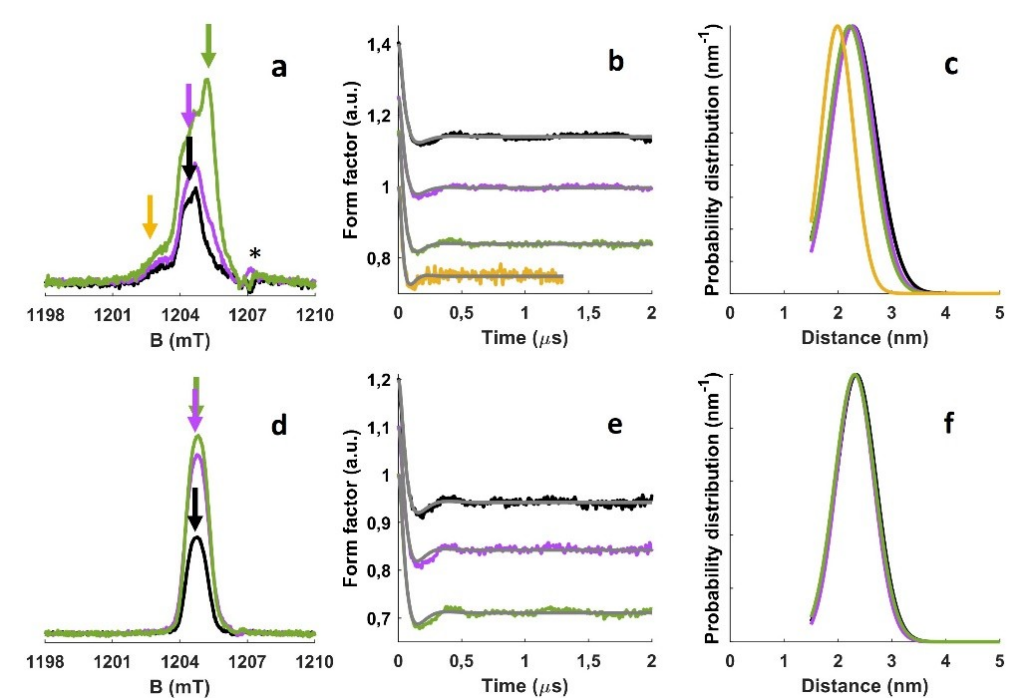
a) Q‐band EDFS spectrum for g‐CN. b) SIFTER experimental form factors for g‐CN and c) corresponding distance distributions obtained through Gaussian fitting. d) Q‐band EDFS spectrum for am‐CN. e) SIFTER experimental form factors for am‐CN and (f) corresponding distance distributions obtained through Gaussian fitting. Colour code: dark (black line), 355 nm (purple line) and 500 nm (green line). The arrows in the EDFS spectra indicate the field positions where the SIFTER measurements were performed: panel (a) black and purple arrows=1204.40 mT, green arrow=1205.00 mT, yellow arrow: 1203.00 mT; panel (d) black, purple and green arrows=1204.75 mT. The asterisk in panel (a) indicates a spurious signal due to the EPR resonator. For better visualization, the SIFTER time traces have been vertically offset. All measurements were performed at 50 K.

Where such paramagnetic species are located within the material, which is the average distance between neighbouring defective sites and how this spatial distribution is affected by light irradiation (i.e. the formation of mobile excitons and trapped electrons and holes) are all questions that have remained so far unanswered. To clarify these points—which are connected to the spatial density of radical species and the photophysics and photochemistry displayed by the material—we exploited the through‐space magnetic dipole coupling between pairs electron spins. Such a coupling is proportional to 1/*r* ^3^, where *r* is the interspin distance, and can be recovered by specific pulsed EPR experiments (Supplementary Figure S7). For narrow and partially overlapping EPR signals, as those reported in Figure 2, the most appropriate PDS sequence is termed SIFTER (single frequency technique for refocusing dipolar couplings) which is based on a solid echo pulse sequence^[33]^ (Supplementary Figure S8). When multiple spin bearing species are present, each individual component can be individually interrogated by adjusting the applied magnetic field. The measured interspin distance can then be compared with the available x‐ray structure to determine the location of the radicals. Figure 4 shows the echo‐detected spectra of g‐CN and am‐CN, the background corrected experimental dipolar evolution time traces (named Form Factors) and the corresponding distance distributions calculated using the DeerAnalysis software^[34]^ assuming a Gaussian distribution (the primary experimental data and a comparison with Tikhonov regularization data processing are reported in Supplementary Figure S9, S10, S11 and S13).

All form factors show a damped oscillation with a sharp initial decay, a minimum at ca. 200 ns after which the data recover and stabilise at a plateau. This characteristic modulation stems from dipolar coupling due to a dominating but distributed distance between spin pairs. The small oscillations visible for evolution times >500 ns are attributed to unsuppressed ^14^N ESEEM contributions, as demonstrated by Supplementary Figure S12. Given that multiple species contribute to the signal of g‐CN, by varying the applied magnetic field, SIFTER experiments were performed at each radical position in dark and after illumination. The dark signal (**1**) yields an average distance of 2.28 nm (width *σ*=0.43 nm). The same radical‐radical distance appears for the sharp and intense signal formed upon irradiation at 500 nm (**3**). On the other hand, the broad radical signal visible at 1203 mT (**2**) is characterised by a significantly shorter and less distributed radical‐radical distance of 1.99 nm with a narrower distribution, *σ*=0.30 nm. The shorter distance obtained for **2** may either be apparent and due to a larger spin delocalisation or reflect a distinct structure and location within g‐CN, as suggested by its unique g‐matrix. The Gaussian fitting of the experimental data for am‐CN (**4**) yields an average radical‐radical distance of 2.34 nm with width *σ*=0.35 nm. Although the signal intensity, and hence the concentration of radical species, increases after illumination both at 355 and 500 nm, the average distance and the distance distribution do not change within the experimental error for all radicals. The width of the distance distributions is related to the microscopic disorder and to the degree of spin delocalisation of the paramagnetic defective states. Given the large modulation depth and the satisfactory fitting obtained through a Gaussian model support the deduction that radical states in CN appear with a regular pattern and that it is not rare to find two radicals at about 2 nm apart. Overall, SIFTER suggests that radical species occur at regular and well‐defined distances both in g‐CN and am‐CN and that the radical‐radical distance depends on the specific radical considered **4**≈**1**≈**3**≠**2**. Furthermore, the data confirm that radical species **1**, **2** and **4** exist as empty, singly and doubly occupied states, since irradiating above or below the band gap result in a higher concentration of species, and that low temperature illumination does not saturate all possible sites, since the average distance between sites is not affected by illumination. By comparing the radical concentration and the number of tri‐*s*‐triazine units we estimate that 1 every 10^5^ units bear an unpaired electron, further confirming that the radical sites are well isolated and sparsely populated. The measured distances for g‐CN and am‐CN under different experimental conditions are all summarised in Table 2.

**Table 2:** Interspin distances as obtained from analysis of the SIFTER data applying a Gaussian model.

Material	Species	Dark		355 nm		500 nm	
		*r* [nm]	*σ* [nm]	*r* [nm]	*σ* [nm]	*r* [nm]	*σ* [nm]
g‐CN	1	2.28	0.43	2.27	0.40		
g‐CN	2	1.99	0.30				
g‐CN	3					2.22	0.39
am‐CN	4	2.34	0.35	2.32	0.35	2.31	0.37

**Figure 5:**
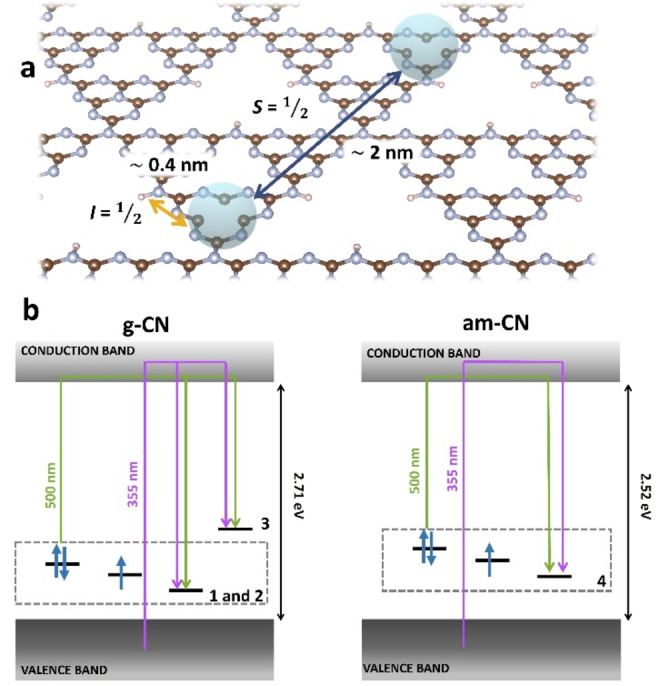
a) Schematic illustration of the local and long‐range structure of radical species in CN. The unpaired electron is confined within a tri‐*s*‐triazine (blue sphere) unit with the closest ^1^H at ca. 0.4 nm (yellow arrow), whereas two closest neighbour radicals are located approximately 2 nm apart (blue arrow). Colour code: brown: carbon; blue: nitrogen. The nitrogen‐vacancy defect depicted in panel (a) provides only a pictorial representation of a plausible spin‐bearing defect in CN. b) Schematic representation of the electronic structure of g‐CN and am‐CN inclusive of defective states. Since they are populated in the dark, defects **1**, **2** and **4** are placed closer to the valence band. Moreover, since their signal intensity increases both under irradiation at 355 and 500 nm, they exist as empty, singly and doubly occupied states. The doubly occupied states are at higher energy because of interelectronic repulsion. Given that it is populated only at low temperature and it shows a tendency to spontaneously decay, defect **3** is positioned at higher energy than **1**, **2**. Purple arrows represent excitation at 355 nm (3.49 eV), whereas green arrows stem from excitation at 500 nm (2.48 eV).

To translate the spectroscopic data into a microscopic description of the system, the distance distributions can be compared with the crystal structure available for g‐CN (entry code: ICSD‐194746). A ≈2.3 nm distance corresponds to the distance between 4 tri‐*s*‐triazine units within a single CN layer, as reported in Figure 5a (see also Supplementary Figure S14), or to ≈7 inter‐layer distances. This establishes the closer proximity between the radical sites that have been implicated as active species in catalysis.^[12]^ Gathering all the data reported, it is possible make inferences on the type and energetics of defective states present and to conclude that both for g‐CN and am‐CN: i) There exists singly occupied defective sites (as demonstrated by the EPR signals in dark); ii) There exist empty and doubly‐occupied sites that trap photo‐generated electrons and holes; iii) The nearest neighbour average distance between radicals does not change upon illumination, therefore photoexcitation at low temperature is not sufficient to saturate all available sites; iv) The radical species are normally distributed within the material as the SIFTER data are satisfactory fitted with a Gaussian model. All these observations are summarised in the energy level scheme for g‐CN and am‐CN reported in Figure 5b, where the relative energy position of the defective states is reported. Additionally, g‐CN presents empty defective states (**3**) that can be populated under light irradiation. Species **3** has been tentatively placed at higher energy than **1** and **2** because it can be trapped only at cryogenic temperature and excitation wavelength <500 nm (see also Supplementary Figure S3) and spontaneously decays.

The spatial distribution of the radical states is pivotal for any catalytic processes based on charge transfer mechanisms. In relation to the C−C coupling towards the synthesis of perfluoroalkylated compounds, DFT modelling attested that only specific spin defects act as anchoring sites for the reagent perfluorobutyl iodide (C_4_F_9_I) and are the active species in the photoinduced charge transfer.^[23]^ Light‐induced paramagnetic species in g‐CN and am‐CN display subtle but not negligible differences. All radicals possess a wavefunction delocalised over a tri‐*s*‐triazine unit, while the nearest neighbour distance, as derived by SIFTER spectroscopy, is consistent with pairs of radical species being separated by 4 tri‐*s*‐triazine units (approximately 2 nm). Unique to g‐CN, a plurality of defective sites with slightly different local structures is stabilised in the crystalline lattice. On the contrary, a single defective site is present in am‐CN, whose local structure is different from all the species present in g‐CN, as revealed by the g‐factors reported in Table 1. It is therefore conceivable that these subtle differences are related to the enhanced photocatalytic activity of am‐CN as compared to g‐CN reported in literature. The high degree of conjugation of CN translates in spin defects with considerable delocalisation which will require careful consideration to fully define the local structure and long‐range distribution of each defect.

## Conclusion

Whether they act as traps promoting charge recombination or are directly involved in catalysis, radical species exert a profound influence in defining the properties of any semiconducting materials. This is even more true for light activated processes that induce a redistribution of charge carriers between valence and conduction bands and all the defective states that lay in the band gap. In this work we show how the structural morphology and light excitation affect the nature and spatial distribution of paramagnetic defective states in CN. The ordered morphology of g‐CN is capable of stabilising at least three paramagnetic defective centers, whereas the less crystalline structure of am‐CN is able to stabilise only a single and characteristic type of paramagnetic centre. Each material responds differently to monochromatic light excitation selectively below or above the band gap. This is a result of the redistribution of photo‐induced charge carriers between the bands and empty and/or doubly‐occupied intragap states. It is worth noting that radical species have been correlated to an augmented catalytic activity towards the perfluoroalkylation of electron‐rich organic substrates. The data reported here clearly show that this is not only due to the number but can also be traced back to the nature of such radical species. The data also demonstrate how the combined use of PDS and hyperfine spectroscopies constitute a novel, independent and powerful methodology to derive the local (sub‐nanometer) and long‐range spatial distribution (nanometer) of paramagnetic species in solid‐state materials with high precision, providing valuable and strong constrains in the interpretation of the photophysics and photochemistry of CN.

## Conflict of interest

The authors declare no conflict of interest.

1

## Supporting information

As a service to our authors and readers, this journal provides supporting information supplied by the authors. Such materials are peer reviewed and may be re‐organized for online delivery, but are not copy‐edited or typeset. Technical support issues arising from supporting information (other than missing files) should be addressed to the authors.

Supporting Information

## Data Availability

The data that support the findings of this study are available from the corresponding author upon reasonable request.
