# Supplementary material for: Morphology and Light‐Dependent Spatial Distribution of Spin Defects in Carbon Nitride
Source: Angew Chem Int Ed Engl. 2022 Sep 27;61(43):e202210640. doi: 10.1002/anie.202210640 (PMC9828381; doi:10.1002/anie.202210640)
Supplement: Supplementary file 1 — Supporting Information [file ANIE-61-0-s001.pdf]

Angewandte Chemie
Eine Zeitschrift der Gesellschaft Deutscher Chemiker

Supporting Information

Morphology and Light-Dependent Spatial Distribution of Spin Defects in Carbon Nitride

A. Actis, M. Melchionna, G. Filippini, P. Fornasiero, M. Prato, E. Salvadori, M. Chiesa*

Supporting information

Contents

EXPERIMENTAL SECTION S2
POWDER XRD SPECTRA S4
EPR SPECTRA DECONVOLUTION S5
ROOM TEMPERATURE CW-EPR SPECTRA S6
EXTENDED HYPERFINE DATA S7
POINT DIPOLE APPROXIMATION e-^{1}H INTERACTION S8
POINT DIPOLE APPROXIMATION e-e INTERACTION S9
SIFTER PULSE SEQUENCE S10
PRIMARY SIFTER DATA S11
^{14}N NUCLEAR MODULATION AVERAGING S13
COMPARISON BETWEEN GAUSSIAN MODEL AND TIKHONOV REGULARISATION ANALYSIS S14
C_3N_4 CRYSTAL STRUCTURES S15
INDEPENDENT REPEATS OF SIFTER DATA S16
REFERENCES S17

EXPERIMENTAL SECTION

Sample preparation. Carbon nitride materials were prepared according to the procedure described in ref. [1]. Briefly, graphitic carbon nitride (g-CN) was obtained from 10 g of melamine heated in a muffle furnace at 550 °C for 300 minutes with a ramping time of 5 °C / min in presence of air. The final product was milled in order to have a uniform powder. To obtain the amorphous sample (am-CN), a certain amount of g-CN was heated in a tubular furnace at 620 °C for 6 hours with a ramping time of 2 °C /min in Ar flow. EPR characterisation was performed on the powder samples without any further treatment. All characterisations were performed keeping the samples in contact with atmosphere.

X-ray powder diffraction. X-ray diffraction patterns were recorded with a PANalytical PW3040/60 X'Pert PRP MPD using a copper Kα radiation source of 0.154056 nm in a Bragg-Brentano geometry. The reflections were scanned continuously with $5° < 2\theta < 80°$. The X'Pert High-Score (Malvern Panalytical Ltd, Malvern, UK) software was used to analyse the patterns.

Diffuse reflectance UV-VIS. The UV–VIS absorption spectra were recorded using a Varian Cary 5000 spectrophotometer (Agilent, CA, USA) equipped with an integration sphere for diffuse reflectance (DR) studies, using the Carywin-UV/scan as software (Agilent, CA, USA). A Teflon sample with 100% reflectance was used as reference.

EPR spectroscopy. Q-band continuous wave and pulse EPR experiments were performed at 50 K on a Bruker ELEXYS 580 EPR spectrometer (microwave frequency ≈ 33.8 GHz) equipped with a Bruker EN 5107D2 resonator, an Oxford Instruments CF935 liquid-helium cryostat and an ITC 503 temperature control unit. The magnetic field was measured by means of a Bruker ER035 M NMR gaussmeter. Irradiation of the samples was performed in the EPR cavity by means of a UV-Vis tunable OPO laser AURORA II (Litron). The laser energy was approximately 50 mW cm^{-2}.

CW EPR. Continuous wave EPR spectra were recorded with a modulation frequency of 50 kHz, a modulation amplitude of 0.1 mT and a microwave power of 0.0063 mW. Simulation of CW EPR data was performed with *Easyspin* [2]. The CW EPR spectra allow to estimate a spin concentration (spin g^{-1}) for g-CN and am-CN of (2.5 ± 0.5) 10^{16} and (5.3 ± 1.0) 10^{16}, respectively.

Echo-Detected Field Sweep (EDFS). EDFS spectra were recorded with the standard Hahn echo sequence π/2−τ−π-echo with π/2 = 16 ns, π = 32 ns and variable τ values (200, 300, 380 and 630 ns) in order to average out the nuclear modulation (ESEEM) effect on the intensity of the echo. The spectra reported in Figure 4 (main text) are the sum over the four τ values.

Hyperfine spectroscopy. 2-pulse ESEEM spectra were recorded with the sequence π/2−τ−π-echo with π/2 = 16 ns, π = 32 ns and starting τ value of 120 ns progressively incremented by 8 ns (Nyquist frequency = 62.50 MHz). Each trace consists of 500 points. Each single trace was then baseline corrected with a 3rd order polynomial function, apodised with a Hamming window function, zerofilled and Fourier transformed (FFT algorithm). ENDOR measurements were performed at 50 K by employing the Mims pulse sequence (π/2- τ - π/2- T- π/2-τ-echo), with π/2 = 16 ns, τ = 216 ns and T = 16 µs. Since the ENDOR spectrum is narrow, the τ value allowed to maximise the signal intensity without distorting the resulting ENDOR spectrum. During the time delay T a RF pulse π_{RF} = 14 µs was accommodated. ENDOR spectra were collected over a 5 MHz sweep with a resolution of 0.02 MHz. HYSCORE measurements performed at 50 K were carried out with the standard pulse sequence π/2–τ–π/2–t1–π–t2–π/2–τ–echo, applying an eight-step phase cycle for eliminating unwanted echoes. Microwave pulse lengths were $t_{\pi/2}$ = 16 ns, t_{π} = 32 ns with τ = 144 ns and the shot repetition rate of was set to 2 ms. The t1 and t2 time intervals were incremented in steps of 20 ns, starting from 120 ns to yield a data matrix of 128 x 128 points. The time traces were then baseline corrected with third-order polynomial, apodized with a Hamming window and zero filled before being transformed into the frequency-domain spectrum through a FFT algorithm.

SIFTER. The deadtime-free four pulse SIFTER (single frequency technique for refocusing dipolar coupling) pulse sequence introduced by Jeschke et al.[3] was used to recover the spatial distribution of radicals. The sequence, which can be summarised as $(\pi/2)_x-\tau_1-(\pi)_x-\tau_1-(\pi/2)_y-\tau_2-(\pi)_x-\tau_2$-echo employed a π/2 = 16 ns and π = 32 ns. The pulse length of 16 ns results in a bandwidth which is sufficient to excite completely the spectrum of a single species. Given the small (if any) anisotropy of the signals, no orientation selection is expected, therefore experiments were conducted at a single magnetic field value for each species. The initial values for τ_1 and τ_2 were both set equal to 1400 ns and the echo evolution was recorded starting 200 ns before zero time. The delay τ_1 was incremented in steps of 4 ns, while τ_2 was decremented by the same amount, yielding an experimental point every 8 ns (corresponding to a Nyquist frequency of 62.50 MHz). In order to remove the strong oscillations (period 200 ns) of the echo intensity due to ^{14}N coupling (ESEEM), an averaging cycle over the nuclear modulation was added to the sequence, in which τ_1 and τ_2 values were both progressively incremented in 8 steps of 50 ns. Therefore the modulation due to electron-nuclear interaction, which depends on $t = \tau_1 + \tau_2$, can be averaged out while maintaining the echo modulation due to electron-electron coupling , which depends on $t = \tau_2 - \tau_1$ [3]. Each trace consists of 335 points. A shot repetition time of 5000 µs was used. A 16-step phase cycle was used. The background decay was fitted to a stretched exponential function $I(t) = e^{-kt^{d/3}}$. Distance distribution was modelled with a Gaussian function using the DeerAnalysis2019 package[4]. Measurements on independent sample preparations are reported in Supplementary Figure S15.

Density of radicals. By comparing the number of radicals obtained through CW EPR data and the number of tri-*s*-triazine units present in the sample (sample mass/molar mass of a tri-*s*-triazine unit) we estimated that 1 every 10^5 tri-*s*-triazine units bears an unpaired electron. The radicals are then well-isolated and the isolated spin pair approximation employed by DeerAnalysis[3] is fulfilled.

POWDER XRD SPECTRA

Powder XRD reveals the typical pattern previously reported in literature for these materials[5] . The two main reflections are 2θ = 12.8°, attributed to the (100) plane and to the intralayer repetition of the tri-*s*-triazine-based motif, and 2θ = 27.5°, ascribed to (002) plane and associated to the interlayer distance. The latter presents a slightly larger linewidth in the amorphous sample, which may be attributed to the partial disruption of the stacked-layer arrangement due to prolonged thermal treatment. From the FWHM of the (002) peak and by using the Scherrer equation a lower bound to the crystallite size can be estimated to be 6.5 ± 0.5 and 5.5 ± 0.5 nm for g-CN and am-CN, respectively.

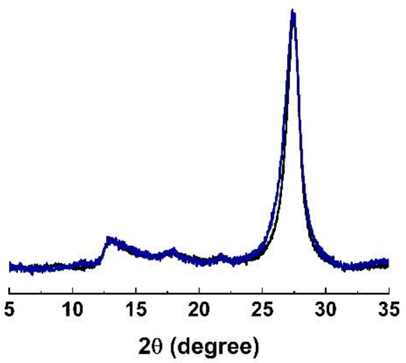

Figure S1: Normalised X-ray diffractograms of g-CN (black) and am-CN (blue) samples.

EPR SPECTRA DECONVOLUTION

The simulation reported in Figure 2 (main text) for g-CN are the sum of the signal attributed to species **1**, **2** and **3**. Figure S2 reports the full deconvolution of the experimental echo-detected spectrum recorded at 50 K showing the individual contribution of each species. The simulation parameters are those reported in Table 1 (main text).

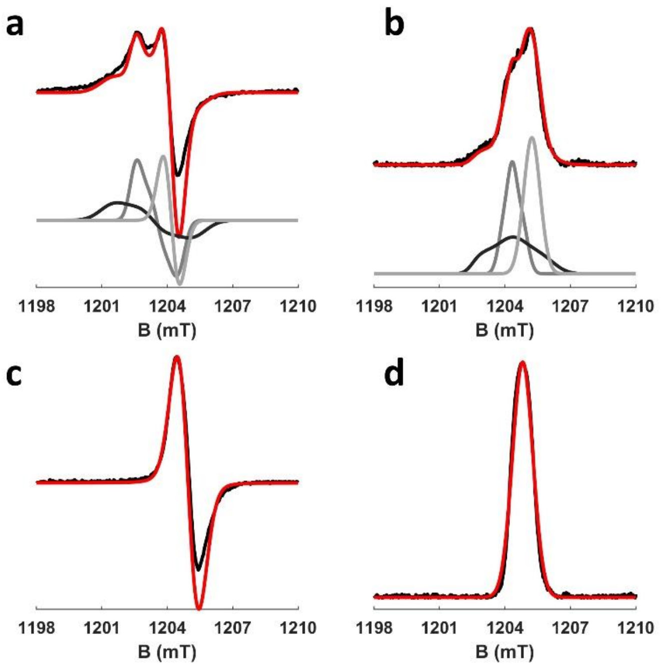

Figure S2: CW (a,c) and EDFS (b,d) spectra recorded on g-CN (top row) and am-CN (bottom row) samples and deconvolution of the simulation to outline the contributions of each individual species. The simulations correspond to the parameters reported in Table 1 (main text).

ROOM TEMPERATURE CW-EPR SPECTRA

The choice to perform EPR measurements at 50 K is twofold. Firstly, it allows to map all trap states even those characterised by lower stability that need low temperature to be efficiently stabilised; and secondly, the relaxation properties of the radicals in carbon nitride require low temperature operation to make PDS viable.

Figure S3 reports the room temperature Q-band EPR spectra for g-CN as a function of the excitation wavelength. Due to its metastable nature, the signal attribute to species **3** is very weak at room temperature but still detectable. Furthermore, from the spectra reported in Figure S3 we estimate that species **3** is efficiently excited only at wavelength < 500 nm, placing a further constraint on its energy position within the bandgap.

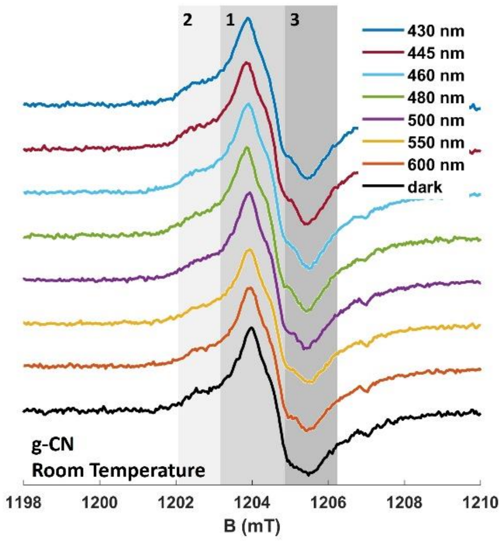

Figure S3: Room temperature Q-band CW EPR spectra for g-CN as function of excitation wavelength. The shaded areas highlight the spectral regions where each species dominates.

EXTENDED HYPERFINE DATA

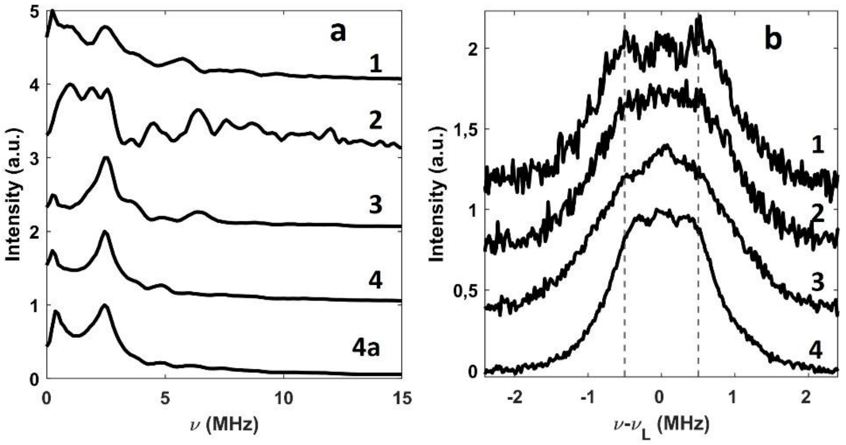

Figure S4: Comparison of all the ^{14}N ESEEM (after FFT) and ^{1}H ENDOR data obtained for the radical species in g-CN (**1**, **2**, **3**) and am-CN (in dark, **4**, and after irradiation at 500 nm, **4**a) at 50 K.

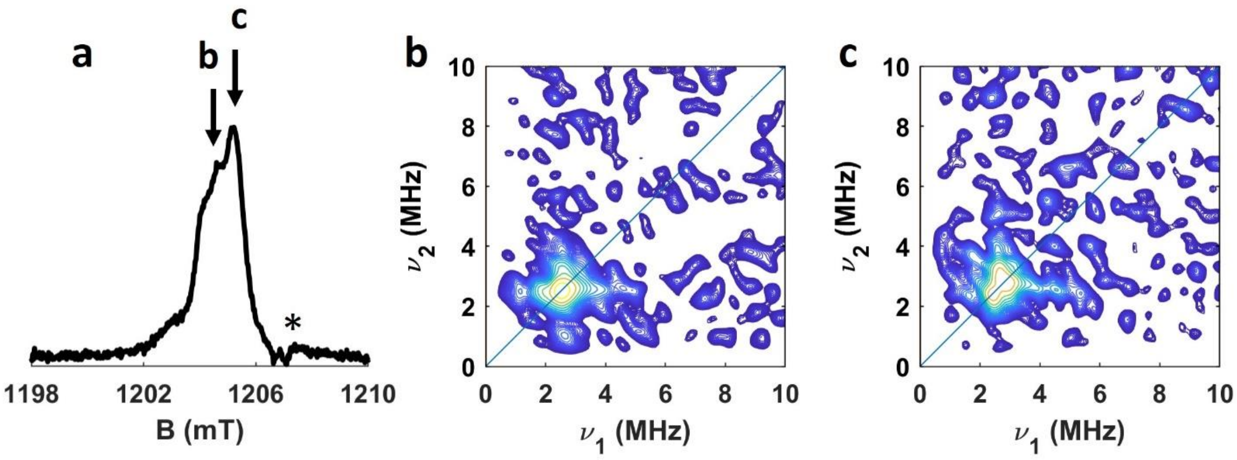

Figure S5: (a) Q-band EDFS spectrum for g-CN. (b) HYSCORE spectrum for species 1 recorded at 1204.4 mT. (c) HYSCORE spectrum for species 3 recorded at 1205.0 mT. All spectra have been recorded at 50 K.

POINT DIPOLE APPROXIMATION e-^{1}H INTERACTION

Under the assumption that the point-dipole approximation holds, the dipolar (axial) contribution *T* to the hyperfine interaction between an electron spin (*S* =1/2) and a nuclear spin (*I*=1/2, e.g. ^{1}H) is inversely proportional to r^3 (the distance between the electron and the nuclear spin) and is given by

$$T = \frac{\mu_0 \beta_e \beta_n g_e g_n}{4\pi\hbar} \frac{1}{r^3}$$

where, μ_0 is the magnetic susceptibility of a vacuum, β_e is the Bohr magneton, β_n is the nuclear magneton of the nucleus under consideration, $\hbar$ is the reduced Planck constant and g_e is the (average) *g*-factors of the electron spins and g_n is the *g*-factors of the nuclear spins.

Therefore, from the dipolar term *T* of the hyperfine coupling the distance between the electron spin and the nucleus can be estimated. For ^{1}H g_n = +5.5856946839 and β_n = +5.050783699 J T^{-1}.

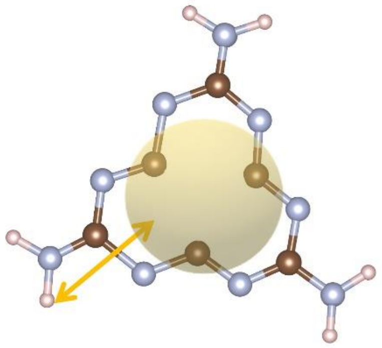

Figure S6: Schematic illustration of the point dipole approximation in the case of ^{1}H-e interaction.

POINT DIPOLE APPROXIMATION e-e INTERACTION

Experimentally, all PDS techniques result in oscillating time traces, where the frequency of oscillation $\omega_{dipolar}$ can be related to the inter-spin distance r according to the equation

$$\omega_{dipolar} = \frac{\mu_0 \beta_e^2 g_{av}^2}{4\pi\hbar} \frac{1}{r^3} (1 - 3cos^2\theta)$$

where, μ_0 is the magnetic susceptibility of a vacuum, β_e is the Bohr magneton, $\hbar$ is the reduced Planck constant, g_{av} is the average g-factors of the two coupled spins, assumed here to be identical, and θ is the angle between the static magnetic field B_0 and r. Given that the bandwidth of the microwave pulse is sufficient to excite the entire spectrum the angular dependence is averaged and $\omega_{dipolar}$ solely depends on r^{-3}.

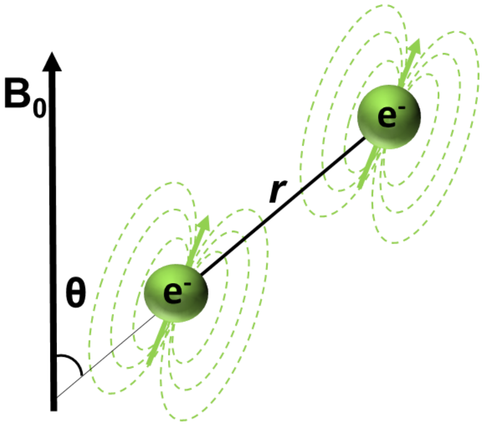

Figure S7: Schematic illustration of the point dipole approximation in the case of e-e interaction.

When distances are distributed, PDS spectroscopy is particularly valuable because the analysis of the experimental data not only provides a single value corresponding to an average interspin distance but also offers the full distance distribution, which defines the probability of finding a pair of spins at a given interspin distance. For unimodal - that is single component - distributions, PDS is extremely sensitive to both the average distance and the width of the distribution[6]. In biological systems the width of the distribution is often discussed in terms of conformational (dynamic) flexibility, however in solid-state systems there is no conformational flexibility and the spread in radical-radical distance (i.e. the width of the distance distribution) provides a measure of the microscopic disorder [7].

In principle, when the dipolar interaction is used to derive distances in delocalised systems, the degree of electron spin delocalization over the surrounding nuclei affects the measured distance and should be taken into account to correct the value obtained from the experiments[8]. Although in structural biology this is a nuisance and strongly localised spin probes are preferred, in the case of CN it is a reporter of the spatial distribution of the wavefunction of the defective states and reports on regions of high spin (and electron) density. In any case, distance measurements between a photoexcited triplet state of a porphyrin (ring diameter ≈ 9 Å [9]) and a nitroxide radical have demonstrated that the spin delocalisation effect is negligible for distances larger than 1.5 nm[10] and likely does not significantly affect the data reported here.

SIFTER PULSE SEQUENCE

The SIFTER (SIngle Frequency TEchnique for Refocusing dipolar couplings) experiment, is a pulse sequence based on the solid echo (Figure S8) able to measure the dipolar coupling between two unpaired electron spins. Since it employs a single microwave source, only moderate spectrometer requirements are necessary.

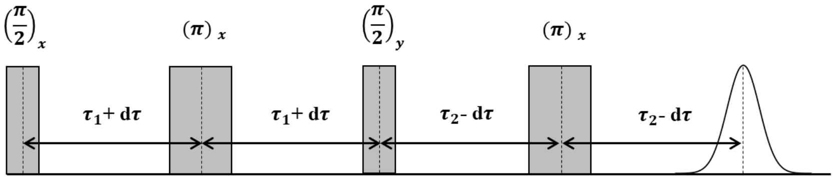

Figure S8: The SIFTER pulse sequence.

The SIFTER echo is measured as a function of τ_2-τ_1 while τ_1+τ_2 is kept constant.

In this work we use rectangular pulses because the bandwidth of the microwave pulse exceeds the spectral width ($\Delta\omega_{sw}$) of the spin system under consideration. This implies that the B_1-field strength associated with the excitation pulse – when given in frequency units by $\omega_1 = \gamma B_1$, (with γ being the gyromagnetic ratio) – is comparable to $\Delta\omega_{sw}$.

PRIMARY SIFTER DATA

Figure 4 (main text) collects and overlays all the SIFTER experiments recorded for g-CN and am-CN. Figure S9 additionally reports the comparison of the primary data (overlaid) before background removal.

As a side note, which is however fundamental to establish PDS as a versatile structural tool, we point out the sensitivity of SIFTER in discriminating small variations in structural features, as it can be readily appreciated by inspecting the inset reported in Figure S9-b that shows the marked effect on the experimental time trace due to a change in the radical-radical distance of ≈ 0.25 nm.

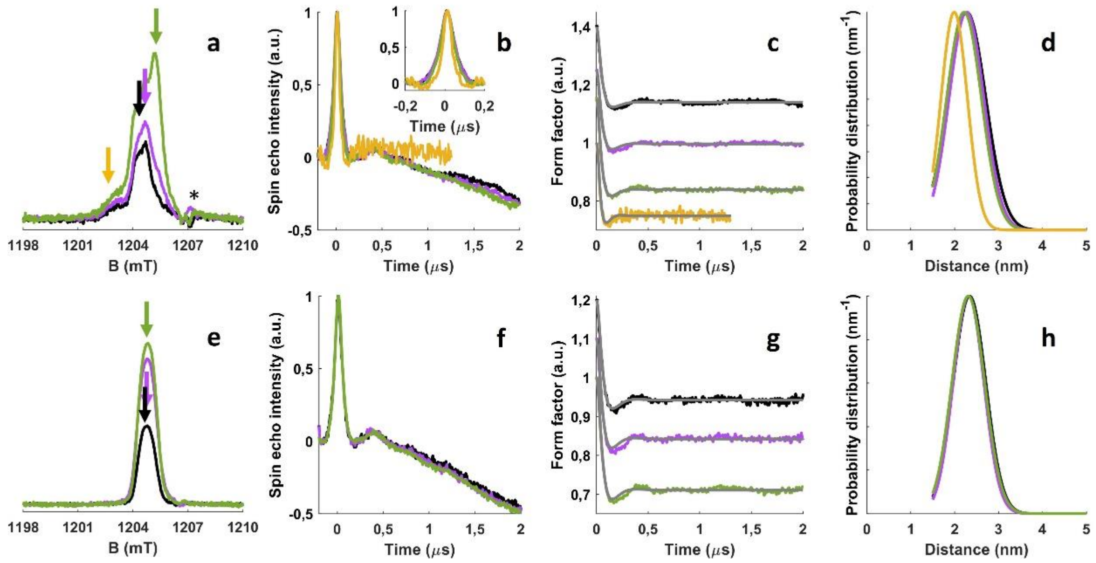

Figure S9: Q-band EDFS spectra (a,e) (the asterisk indicates a spurious signal), normalised primary SIFTER data (b,f), experimental form factors as obtained after DeerAnalysis data processing (c,g) and distance distributions (d,h) obtained through Gaussian fitting of g-CN (a) and am-CN (e) at different irradiation conditions: dark (black line), 355 nm (purple line) and 500 nm (green line). The arrows in the EDFS spectra indicate the field positions where the SIFTER measurements were performed. For better visualization, the SIFTER time traces have been vertically offset.

Figures S10 and S11 show the individual primary data (before the background fitting and removal) as well as the fitting based on a Gaussian model and the corresponding distance distribution for g-CN and am-CN, respectively.

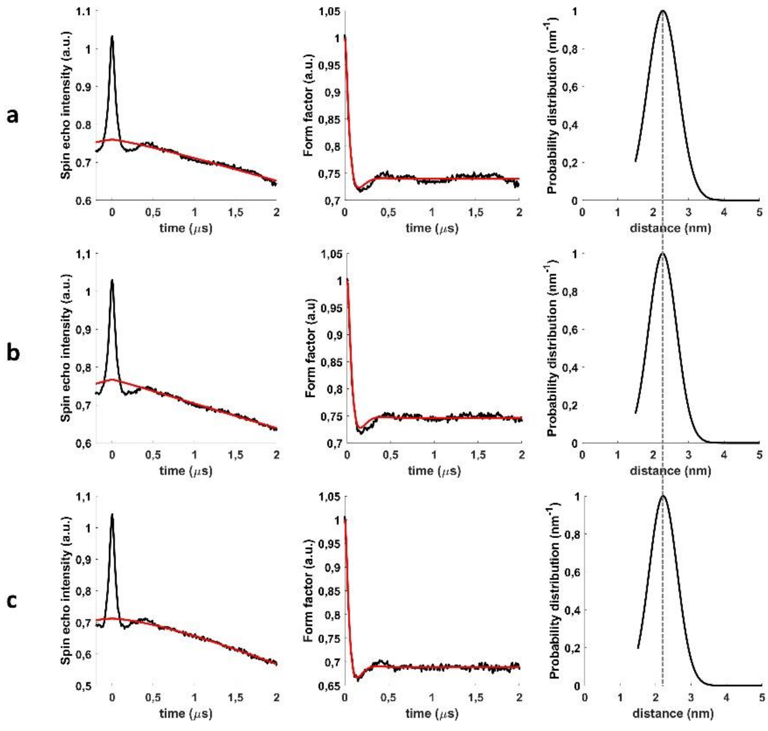

Figure S10: SIFTER primary data for g-CN sample (black) with stretched exponential background (red), experimental form factor (black) and corresponding fitting (red) and corresponding distance distributions (DeerAnalysis2018) recorded in dark (**a**, species **1**) and under irradiation at 355 nm (**b**, species **1**) and 500 nm (**c**, species **3**).

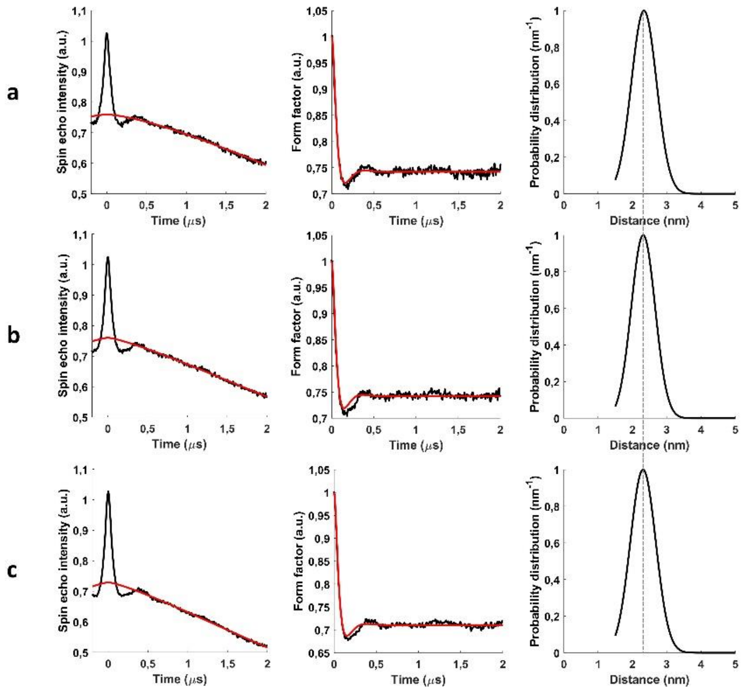

Figure S11: SIFTER primary data for species **4** in am-CN sample (black) with stretched exponential background (red), experimental form factor (black) and corresponding fitting (red) and corresponding distance distributions (DeerAnalysis2018) recorded in dark (**a**) and under irradiation at 355 nm (**b**) and 500 nm (**c**).

^{14}N NUCLEAR MODULATION AVERAGING

It is known that the SIFTER experiment may be contaminated by nuclear modulation effect (a.k.a. ESEEM effect), which may complicate the analysis and the reliability of the outcomes. However, while the electron-electron dipolar modulation depends on τ_2-τ_1, the electron-nuclear modulations depend on τ_1+τ_2. For this reason, 8 independent SIFTER differing for the total length of the experiment where collected and summed. The positive averaging effect of this procedure is illustrated by Figure S12, which compares the SIFTER experiments recorded for species **4** in am-CN with and without nuclear modulation averaging.

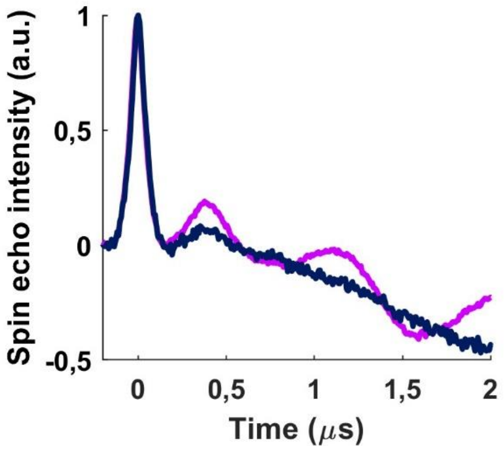

Figure S12: Normalised SIFTER data for am-CN sample with (blue) and without (violet) nuclear modulation averaging.

COMPARISON BETWEEN GAUSSIAN MODEL AND TIKHONOV REGULARISATION ANALYSIS

In order to confirm that robustness of the Gaussian model fitting and that the additional modulations visible at t > 500 ns are due to electron-nuclear hyperfine interaction it was compared against Tikhonov regularisation analysis. Figure S13 reports the comparison between the Gaussian model and the Tikhonov regularisation analysis for species **1** and **4**. For this comparison the background dimensionality was kept fixed to 3 and a regularisation parameter, α, equal to 100 was used.

From inspection of Figure S13, Tikhonov regularization splits the average distance obtained from the Gaussian model into two discrete distances while keeping the total width almost unchanged. In any case, even Tikhonov regularization is not able to account fully for the additional modulations, confirming that they originate form electron-nuclear hyperfine interaction.

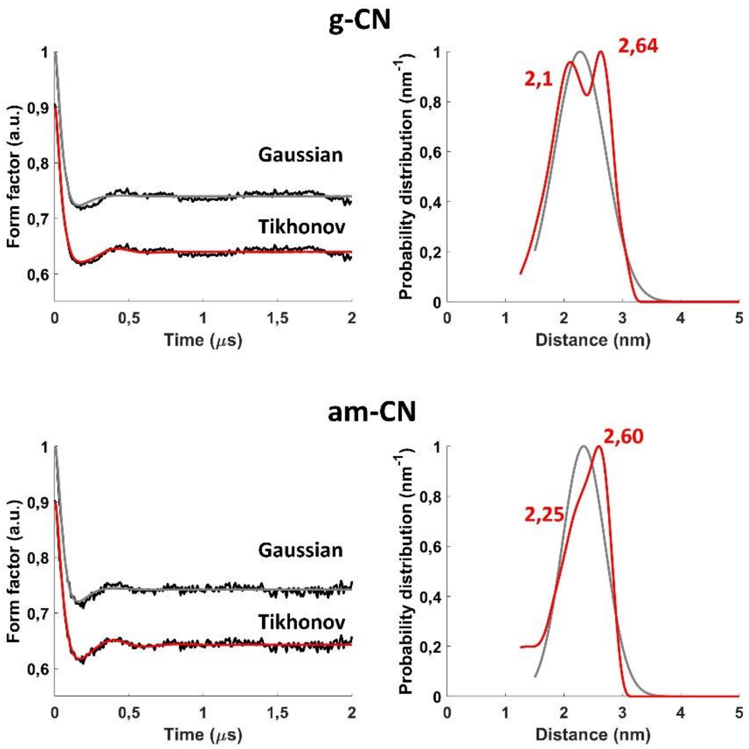

Figure S13: Comparison between the Gaussian model fit and the Tikhonov regularisation analysis for species **1** in g-Cn(top) and species **4** in am-CN (bottom). For ease of comparisons, the form factors have been vertically translated.

C_3N_4 CRYSTAL STRUCTURES

Two main crystal structures have been reported for the stoichiometry C_3N_4, the first corresponds to an extended network of fully polymerised molecular precursors (discussed in the main text, entry code: ICSD-194746) whereas the second corresponds to a partially polymerised arrangement of molecular precursors (entry code: ICSD-194747). For completeness, Figure S14 provides a comparison between the spectroscopically derived distances overlaid on the two structures. In both cases the double-headed arrow corresponds to a distance of ≈ 2.3 nm.

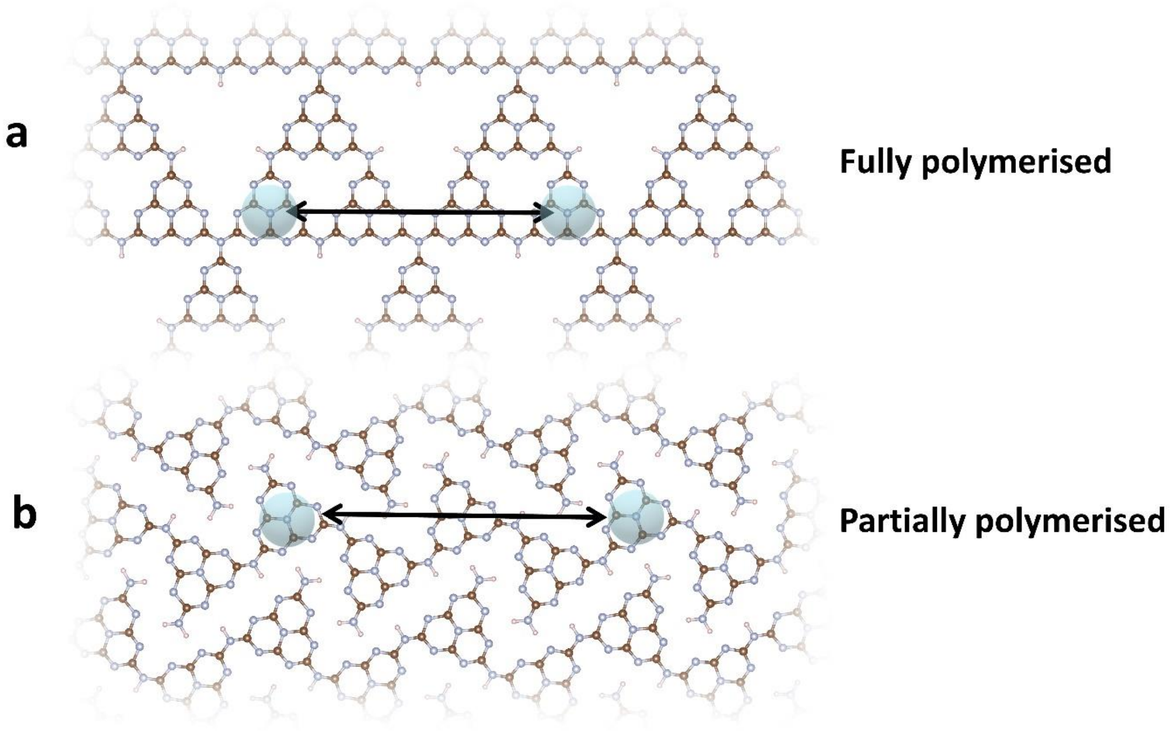

Figure S14: Schematic illustration of the long-range structure of radical species in CN considering the two available crystal structures. The unpaired electron is confined within a tri-*s*-triazine (blue sphere). The closest neighbour radicals are located approximately 2.3 nm apart (double-headed arrow). Colour code: brown: carbon; blue: nitrogen.

INDEPENDENT REPEATS OF SIFTER DATA

To confirm that the results obtained and presented in the manuscript are general and representative of the CN materials synthesised according to the experimental procedure reported in the experimental section, we performed SIFTER measurements on independent sample preparations. This is a good practice that has been recently recommended for PDS experiments on biomolecules[11].

Figure S15 compares the SIFTER time traces for species **1** in g-CN and **4** in am-CN. The data reported in Figure S15 assures that the applied methodology leads to reproducible distance distributions, which are not batch dependent.

Repeats of the SIFTER experiments on the same sample after freezing-thawing cycles or after illumination-thermal annealing always gave consistent and fully reproducible result.

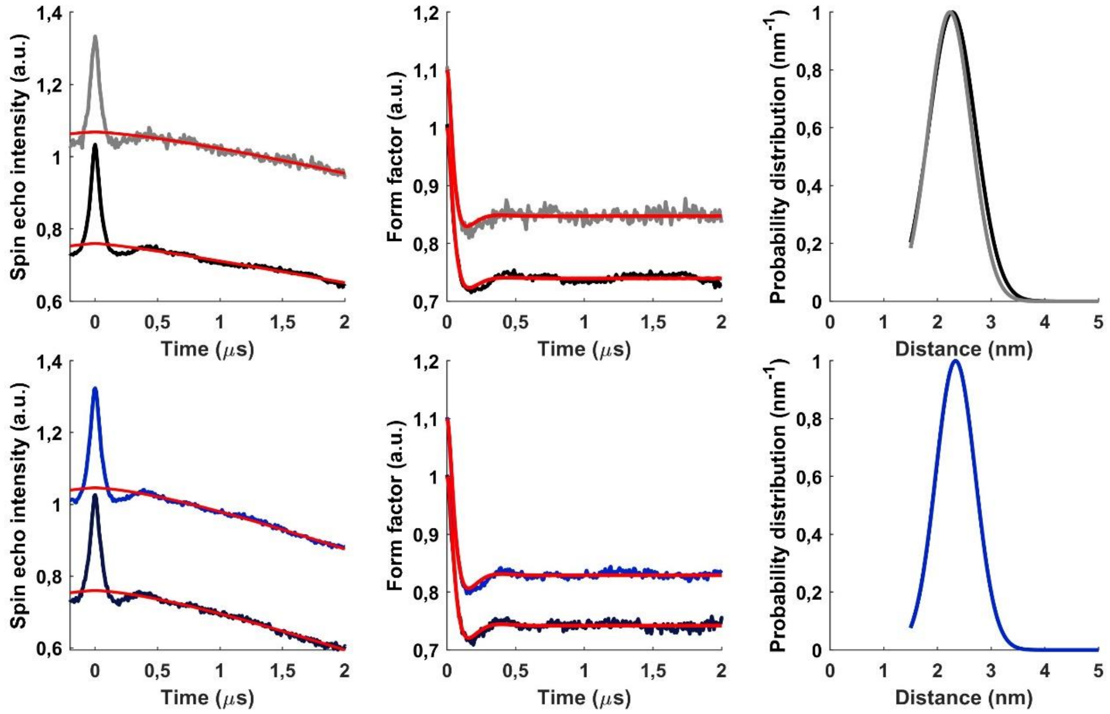

Figure S15: SIFTER primary data for g-CN (black and grey) and am-CN (dark and light blue) samples recorded on two different synthesis batches. Distance distributions are obtained through a Gaussian model.

REFERENCES

[1] F. Longobardo, G. Gentile, A. Criado, A. Actis, S. Colussi, V. Dal Santo, M. Chiesa, G. Filippini, P. Fornasiero, M. Prato and M. Melchionna, *Mater. Chem. Front.*, **2021**, *5*, 7267-7275

[2] S. Stoll and A. Schweiger, *J. Magn. Reson.,* **2006**, 178(1), 42-55

[3] G. Jeschke, M. Pannier, A. Godt and H.W. Spiess, *Chem. Phys. Lett.*, **2000**, *331*, 243-252

[4] G. Jeschke, V. Chechik, P. Ionita, A. Godt, H. Zimmermann, J. Banham, C.R. Timmel, F. Hilger and H. Jung, *Appl. Magn. Res*. **2006**, *30*, 473–498

[5] F. Fina, S. K. Callear, G. M. Carins, J. T. S. Irvine, *Chem. Mater*., 2015, **27** (7), 2612–2618

[6] R. A. Stein, A. H. Beth, E. J. Hustedt, *Meth. Enzymol*., 2015, **563**, 531-567

[7] E. Salvadori, E. Fusco and M. Chiesa*, J. Phys. Chem. Lett.*, 2022, **13** (5), 1283–1289

[8] A. Collauto, A. Feintuch, M. Qi, A. Godt, T. Meade, D. Goldfarb, J. Mag. Res., 2016, 263, 156-163

[9] N.V. Suendo, A. Alni, A. A. Nugroho, Y. Majima, S. Lee, Y. P. Nugraha and Hidehiro Uekusa, *J. Phys. Chem. A*, 2020, **124** (13), 2672–2682

[10] M. Di Valentin, M. Albertini, M. G. Dal Farra, E. Zurlo, L. Orian, A. Polimeno, M. Gobbo, D. Carbonera, *Chem. Eur. J.* 2016, **22**, 17204 – 17214

[11] O. Schiemann, C. A. Heubach, D. Abdullin, K. Ackermann et al., *J. Am. Chem. Soc.*, 2021, **143**, 17875−17890
